# Treatment of Croup

**Published:** 1885-06

**Authors:** F. E. Waxham

**Affiliations:** Professor of Diseases of Children, College of Physicians and Surgeons, of Chicago; 3449 Indiana Avenue


THE CHICAGO

### MEDICAL JOURNAL AND EXAMINER.

VOL. L. JUNE, 1885. No. 6.

### ORIGINAL COMMUNICATIONS.

ARTICLE I.

TREATMENT OF CROUP. *By* F. E. WAXHAM, M. D., *Professor of Diseases of Children, College of Physicians and Surgeons, of Chicago.*

(Read before the Chicago Medical Society, April 20, 1885.)

In the treatment of pseudo-membranous laryngitis a host of specifics have been proposed from time to time. The fatality of the disease is so great that it is not strange that so many different remedies have been advocated. Some place their faith in emetics, others in veratrum viride, others in mercury, still others in various inhalations, and so on until almost the whole catalogue of remedies has been exhausted. It is undoubtedly true that we possess no single remedy that can be depended upon with which to successfully treat this disease, even in a small proportion of cases. Neither will any one line of treatment be appropriate in every case. In many cases the danger is from asthenia. If we can only keep up the strength, the child will struggle through. In these cases, iron, quinine, nourishment and stimulants, are our most important agents. In other cases the great danger is from suffocation, and no amount of strength would be of avail, unless we can clear the larynx of the false membranes, or make an artificial opening through which respiration may be conducted.

In other cases we are not called until the child is upon the verge of suffocation, and tracheotomy, or intubation of the larynx, is our only resort.

In other cases, again, we are called early, and oftentimes witness the slow, unrelenting progress of the disease in spite of our most earnest efforts to arrest it.

Not only should the treatment vary in different cases, but in the different stages of the same case. We shall be much more successful in the treatment of this disease if we meet the various indications as they arise, than if we depend upon any single remedy. Among the most important indications we may enumerate the following:

- 1st. Prevent as far as possible the formation of false membrane.
  - Cold applications,
  - Blister,
  - Quinine,
  - Digitalis,
  - Aconite,
  - Veratrum,
  - Emetic,
  - Purgative.
- 2d. When formed cause its dissolution and expulsion as quickly as possible.
  - Spray.
    - Pancreatin,
    - Lime water,
    - Liq. potassæ,
    - Lactic acid,
    - Phosphoric acid.
  - Bichloride of mercury,
  - Lactate of iron,
  - Emetics.
- 3d. Control laryngeal spasm.
  - Opium,
  - Bromide of ammonium.
- 4th. Prevent complications — Uniform temperature.
- 5th. Support the strength of patient
  - Nourishment,
  - Stimulants.
- 6th. In case of threatened suffocation, tube the larynx or perform tracheotomy.

Among the agents for meeting the first indication, the prevention of the plastic exudation, the greatest confidence has been placed in cold applications, blisters, an emetic or purgative and quinine, digitalis, aconite or veratrum.

As the disease is generally conceded to be a local inflammation, and the membrane an exudation resulting therefrom, it is certainly clear that so far as we are successful in controlling the local inflammation, so far will we be successful in limiting its product, the exudate. Hence the benefit from cold applications, as advised by Professor Peaslee, and the use of the blister, in which Professor Earle, of this city, has much confidence; the use of veratrum viride and emetics, as advised by Professor Fordyce Barker, or in its place digitalis, or aconite if there is danger from asthenia; for the same reason, large and frequent doses of quinine are indicated.

To meet the second indication, to cause the dissolution and expulsion of the false membrane, we must resort to solvents and emetics. From various experiments with different solvents I am satisfied that trypsin is the quickest and most satisfactory. Next in order, lime water, then liquor potassæ, and then ten per cent. solution of lactic and dilute phosphoric acids. These solvents should be used in the form of spray from an atomizer.

Dr. Henry Dwight Chapin, in a very able article published in the *Medical Record*, March 7, 1885, advocates the use of trypsin, and gives the following formula:

℞

Fairchild's Ext. Pancreatis.................. gr. xv.

Sodii Bicarb.................................. gr. iij.

Glycerin...................................... ʒj.

Aq. Dest...................................... ʒvij.

M.

My experiments confirm entirely the value of this combination. I prefer, however, using equal parts of glycerine and water as the menstruum.

A very important consideration referred to by Dr. Chapin is the proper form of atomizer. As used in the ordinary hand atomizer these solutions are of little benefit, as it is a question as to how much of the solution reaches the larynx. The atomizer, as recommended by Dr. Chapin, should terminate in a rounded nozzle, that should be directed downward at a right angle to the stem of the atomizer. This can be thrust far backwards in the mouth without fear of injury, and the spray thus directed into the larynx. These solutions, to be of benefit, must be used frequently, at least every half hour.

In regard to lime water, it is easily proven to be one of our quickest solvents of false membrane. Time and again I have seen false membrane dissolved in an officinal solution of lime water, in from seven to twenty minutes. While lime water is second only to trypsin in its solvent power, yet the vapor of lime, in whatever form, is useless as a solvent. At one time I was somewhat enthusiastic over the inhalation of the vapor of lime in the treatment of croup, and exhausted considerable time and ingenuity in perfecting suitable apparatus for its convenient administration. Finally, after several cases terminated fatally, although they were nearly smothered with it, I became skeptical, and am now fully convinced that it possesses no solvent power. Whatever benefit is to be derived from it is due simply to the warmth and moisture.

A two per cent. solution of liquor potassæ, and ten per cent. solutions of lactic and phosphoric acids, are less active solvents than either officinal lime water or trypsin.

Of the internal remedies for promoting disintegration and separation of the false membrane, perhaps those that have received the highest sanction are lactate of iron and bichloride of mercury. I have never used the lactate of iron being discouraged by the slow digestive action of lactic acid. The bichloride of mercury I have used thoroughly in five cases' both before and after tracheotomy, only one case terminating favorably. In this case the favorable termination was ascribed to other treatment as well. For causing the rejection of the false membrane almost every emetic has been employed and advocated. I am satisfied that alum, or alum combined with ipecac, is as good as any.

The third indication, the relief of the laryngeal spasm, is best met by the use of opium or bromide of ammonium. Opium serves a double purpose, as it relieves pain as well as spasm.

The patient should be supported by the most nourishing diet, and by stimulants if necessary.

Complications will be avoided in a great measure by keeping the room at a uniform temperature. In case of threatened suffocation it is our plain duty either to perform tracheotomy or tube the larynx.

A desperate case of croup has just come under my observation in a child four years old, and a brief history may not be uninteresting.

The disease commenced in the form of nasal diphtheria, with considerable inflammation and swelling of the tonsils. There was a profuse muco-purulent discharge from the nostrils, and the breathing nasal in character. After one week a slight, thin membranous exudation appeared upon the tonsils, and the next day a croupy cough occurred. Respiration became gradually embarrassed, the dyspnœa increased and became urgent, and the respiration characteristic of pseudo-membranous laryngitis. The cough gradually became more and more husky, and the voice whispering. The pulse ranged at about 120 to 130; temperature, 101° F.

*Treatment.*—Quinine, gr. 1¼, every 2 hours, alternating with gtt. ij. tr. chloride of iron, combined with chloride of potash, glycerine and simple syrup, vapor of lime from a kettle over an oil stove constantly, and the following mixture by means of the hand atomizer:

℞

Fairchild's Ext. Pancreatis..................gr. xxx.
Sodii Bicarb.................................... gr. vj.
Glycerin ............ ..... .......................
Aq. Dest.......................................... aa. ℥j
M.

A large blister was also applied over the region of the larynx. On the third day after the occurrence of the croupy cough, the respiration being difficult, the voice and cough suppressed, the iron mixture was omitted, and $\frac{1}{24}$ gr. of bichloride of mercury given every hour. Quinine and other treatment continued. Warm poultices covered with oil silk were also applied to the chest.

Within forty-eight hours after the administration of the mercury a number of large masses of false membrane were rejected, measuring from 1½ to 2 inches in length by ½ inch in width, with immediate relief to the urgent dyspnœa.

False membrane was occasionally rejected in small masses for the next two or three days.

On the third day after the first rejection of false membrane, the temperature was suddenly elevated to 104° F., respiration, 20; pulse, 160, and very feeble.

The child was greatly prostrated and covered with a fine rash closely resembling the eruption of scarlet fever, causing the child much annoyance from the intense itching. The cough, short and painful. Physical examination revealed a broncho-pneumonia. The bichloride of mercury, which had been given less frequently after the expulsion of the false membrane, was now discontinued, but the quinine was continued, alternating with tr. digitalis, combined with carbonate of ammonium and syr. of squills, giving each every four hours.

The cough within a day or two became loose and less painful, and the sputa characteristic of pneumonia. There was no recurrence of the membrane within the larynx, the respiration continuing unembarrassed, but the voice remained weak, and more or less whispering, for several days. The child made a good but gradual recovery.

In this case several masses of false membrane were rejected at different times. Four pieces, each measuring one and a half inches in length by one-half inch in width, were subjected to the following experiments :

1st. One of these masses of membrane was pinned to a board ten inches from the spout of a kettle containing a turbid solution of lime water, and heated by an oil stove. For three hours a constant stream of steam was poured forth upon this membrane without any perceptible effect.

The membrane was not softened or disintegrated in the least.

2d. The membrane that had been subjected to the steaming process without any seeming effect was placed in a two ounce vial containing an officinal solution of lime water. In seven minutes it was entirely and completely dissolved.

3d. Another mass of membrane was attached to a cork, and subjected every half hour to a spray from a hand atomizer, of the following solution :

℞

Fairchild's Ext. Pancreatis....................gr. xv.
Sodii Bicarb ....................................gr. iij.
Glycerin ...........................................ʒss.
Aquæ ..............................................ad. ℥j.
M.

In one hour and a half, and after four applications of the spray, the membrane was entirely disintegrated.

4th. Another mass of equal size was subjected in the same manner, to a spray of officinal lime water. In two hours and a half, and after six applications of the spray, the membrane was completely dissolved.

5th. Still another mass of membrane of the same size and apparent consistency, was subjected under exactly the same circumstances to a spray of a ten per cent. solution of lactic acid. In three hours and a half the membrane was somewhat softened, but not disintegrated and completely softened as in the previous cases.

The conclusions to be deduced are:

1st. A solution of pancreatin, with soda and glycerine, is the quickest and most satisfactory solvent.

2d. Lime water in the form of spray is a rapid solvent of false membranes, but does not act as quickly as a solution of pancreatin.

3d. The vapor of lime, however thoroughly applied, is entirely useless as a solvent.

4th. Lactic acid is not a satisfactory solvent.

If I may be permitted to still further encroach upon your time and patience, I would refer briefly to tubing of the larynx.

I am convinced that the profession in Chicago, while they sanction tracheotomy, are not enthusiastic over it, and that they will gladly accept any substitute and give it a fair trial. Such a substitute has been proposed in the tubing of the larynx. To Dr. O'Dwyer, of New York City, belongs the great credit of originating this bold and ingenious method of treating croup. While the larnyx has been tubed, from time to time, for laryngeal obstructions, yet none were bold enough to use tubes of sufficient length to drop into the trachea and to leave them in situ. Although occasionally performed, the method was entirely different from that advocated and practiced by Dr. O'Dwyer. To Dr. O'Dwyer belongs the entire credit of tubing the larynx, as will be illustrated to-night.

We can see no objection that can be raised to this method of treating croup that does not apply to tracheotomy as well. True, the false membrane may extend below the tube and cause obstruction in the trachea, but this is just as likely, or even more likely, to occur after a tracheotomy.

The advantages of intubation are:

1st. That with a little dexterity and practice it can be very easily and quickly performed and without danger.

2nd. We have no mutilation or disfigurement of the patient.

3rd. We have no wound that may be the cause of shock or that may be the source of systemic infection.

4th. The laryngeal tube can be worn much more easily, with less irritation, than the canula after a tracheotomy. Coughing and expectoration occur just as easily.

5th. It does not require the close and unremitting care of the medical attendant, as does a tube after tracheotomy.

6th. The air that reaches the lungs is warm from its contact with the upper air passages, and not as likely to cause bronchitis or pneumonia.

7th. Much less objection will be raised by parents against tubing.

As these instruments have been but recently obtained, my experience is limited to a single case, which may not be uninteresting.

On Sunday last, April 19th, I was called to attend a little child two years and one month old, suffering from true croup. The child had been sick for two or three days, and respiration had been very difficult during the whole of the previous night.

Upon examination, false membrane was observed upon both tonsils, which were considerably swollen and inflamed. The cough was husky and frequent, and the voice whispering. The pulse very rapid and feeble. The respiration was labored, there being great difficulty in expiration as well as inspiration. The face deeply flushed and covered with perspiration, while with every inspiration there was depression of the soft tissues at the base of the sternum and underneath the clavicles.

An emetic was administered and considerable ropy mucus expelled, but with little relief to the urgent dyspnœa.

At 1 P. M., Sunday afternoon, the child was in a comatose condition, the head thrown far backward, the features of a deadly pallor, the pulse feeble and rapid, and the respiration difficult in the extreme. The child was in the last stages of suffocation. In this condition the larynx was tubed without difficulty and without resistance, and with immediate relief to the urgent symptoms. The breathing at once became easy and tranquil, the natural color returned to the lips and face, and the child soon fell into a quiet sleep.

At 5 P. M. the patient was seen by Dr. Steele as well as myself. Respiration, 25 per minute; pulse, 120, and of good strength; temperature, 103.8° F. The little patient seemed bright, and interested in what was going on around him. He ate half a cup of soft bread moistened with milk, eagerly, and then teased for candy. Water and milk whenever given provoked coughing, but soft bread could be easily swallowed. The patient was given four grains of quinine every two hours, per rectum, and frequently bathed with cool water.

9 P. M. Respiration, 48; pulse, 125; temperature, 103° F. The patient bright and inclined to be playful.

1:30 A. M. Tube rejected after remaining in position twelve hours. The child was seen one hour later, when the symptoms being again urgent, the tube was replaced with immediate relief. Pulse, 130; respiration, 32; temperature, 101½° F.

April 20th, 9 A. M. Pulse, 130; temperature, 101°F.; respiration, 44. The respiration now indicated extension of false membrane downward. It was becoming more labored and rasping in character.

1 P. M. Respiration very much embarrassed and the child becoming very restless and livid in color. The tube was now removed, and as respiration was as difficult with as without it, its further use was abandoned. The child died at 7 P. M., thirty hours after intubation.

We cannot judge of the value of any operation from a single case. We must withhold our judgment until a considerable number of cases have been recorded, and only until then can we determine the value of intubation of the larynx. We certainly should feel very hopeful that it will soon be considered a legitimate operation.

3449 Indiana Avenue.

